# Solvent Microenvironments and Copper Binding Alters the Conformation and Toxicity of a Prion Fragment

**DOI:** 10.1371/journal.pone.0085160

**Published:** 2013-12-27

**Authors:** Mohammed Inayathullah, K. S. Satheeshkumar, Andrey V. Malkovskiy, Antoine L. Carre, Senthilkumar Sivanesan, Jasper O. Hardesty, Jayakumar Rajadas

**Affiliations:** 1 Biomaterials and Advanced Drug Delivery Laboratory, Stanford University School of Medicine, Stanford, California, United States of America; 2 Bioorganic and Neurochemistry Laboratory, Central Leather Research Institute, Adyar, Chennai, India; 3 Department of Surgery, School of Medicine, Stanford University, Stanford, California, United States of America; 4 Department of Chemical Engineering, Stanford University, Stanford, California, United States of America; 5 Department of Neurology and Neurological Science, Stanford University School of Medicine, Stanford, California, United States of America; 6 Cardiovascular Pharmacology Division, Cardiovascular Institute, Stanford University School of Medicine, Stanford, California, United States of America; Van Andel Institute, United States of America

## Abstract

The secondary structures of amyloidogenic proteins are largely influenced by various intra and extra cellular microenvironments and metal ions that govern cytotoxicity. The secondary structure of a prion fragment, PrP(111-126), was determined using circular dichroism (CD) spectroscopy in various microenvironments. The conformational preferences of the prion peptide fragment were examined by changing solvent conditions and pH, and by introducing external stress (sonication). These physical and chemical environments simulate various cellular components at the water-membrane interface, namely differing aqueous environments and metal chelating ions. The results show that PrP(111-126) adopts different conformations in assembled and non-assembled forms. Aging studies on the PrP(111-126) peptide fragment in aqueous buffer demonstrated a structural transition from random coil to a stable β-sheet structure. A similar, but significantly accelerated structural transition was observed upon sonication in aqueous environment. With increasing TFE concentrations, the helical content of PrP(111-126) increased persistently during the structural transition process from random coil. In aqueous SDS solution, PrP(111-126) exhibited β-sheet conformation with greater α-helical content. No significant conformational changes were observed under various pH conditions. Addition of Cu^2+^ ions inhibited the structural transition and fibril formation of the peptide in a cell free *in vitro* system. The fact that Cu^2+^ supplementation attenuates the fibrillar assemblies and cytotoxicity of PrP(111-126) was witnessed through structural morphology studies using AFM as well as cytotoxicity using MTT measurements. We observed negligible effects during both physical and chemical stimulation on conformation of the prion fragment in the presence of Cu^2+^ ions. The toxicity of PrP(111-126) to cultured astrocytes was reduced following the addition of Cu^2+^ ions, owing to binding affinity of copper towards histidine moiety present in the peptide.

## Introduction

The highly infectious and transmissible prion disease occurs due to the misfolding and aggregation of prion proteins, resulting in neurodegeneration [1,2,3,4]. The misfolded form of prion (PrP^Sc^) have been shown to be the causative agent in numerous neurodegenerative disorders, including Cruetzfeldt-Jakob disease (CJD), Gerstmann-Straussler disease (GSD) and fatal familial insomnia (FFI) in humans, as well as Scrapie in sheep, and bovine spongiform encephalopathy in cows [5,6]. PrP^Sc^ has been found to self-assemble into fibrillar aggregates/amyloids that accumulate in the nervous system, often leading to neuronal death [7]. Prionoids is the term recently coined for the pathological aggregates of various amyloid proteins that can infect neighboring cells and propagate amyloidosis [3,8]. Prion mediated neurotoxicity mechanisms involve the accumulation of oligomers in aggresomes [9,10,11], proteosomal dysfunctions [12,13], apoptotic mechanisms [10], triggering of endoplasmic reticulum mediated apoptotic cascades [14], microglial activation [15,16], increased phosphorylation of elF2-α levels [17], calpains mediated toxicity [18], and dysfunction of PI3K/AKT/GSK signaling [19]. 

Although the precise function of native/cellular form of prion protein (PrP^C^) in healthy tissues is not known, earlier research demonstrates that it binds Cu^2+^ in an unusual and highly conserved region of the protein termed the octarepeat domain [20,21,22,23]. Copper has diverging functions on prion kinetics based on *in vivo* and *in vitro* conditions, either by supporting or suppressing amyloid aggregation events [24,25]. Conversely, imbalance of redox-iron homeostasis is clearly manifested in prion infected cells, attributed towards prion disease pathogenesis [26,27]. However, key mechanisms that govern the transformation of α-helical rich PrP^C^ to a β- sheet rich PrP^Sc^ is not well understood. The gross secondary structure of PrP^Sc^ is known to contain a higher β-sheet content than PrP^C^ [28]. A model for the tertiary structure of PrP^Sc^ has also been suggested [29]. Further, a parallel β-helical structure for PrP^Sc^, based on electron microscopy work has been proposed [30]. 

Previous conformational studies indicate that a transition from α-helical to β-sheet structures (PrP^C^ PrP^Sc^) is likely to be the crucial event in prion propagation [31,32,33,34,35]. To investigate which portions of the native protein sequence are involved in the conformational transition from PrP^C^ to PrP^Sc^ and PrP amyloid, several groups have analyzed the secondary structures and fibrillogenic properties of synthetic PrP peptides [36]. Extensive work with these peptides has established that the glutamine-rich consecutive segment of PrP that spans residues 106-147 is important for deciphering the fibrillogenic properties of protein [37,38,39]. In particular, the sequence encompassing residues 106-126 of human prion protein was convincingly found to be neurotoxic [37]. This segment corresponds to a highly conserved region of PrP located towards the N-terminus and adjacent to the stable globular domain that does not misfold [40]. PrP(106-126) consists of an N-terminal polar head (KTNMKHM) with a long hydrophobic tail (AGAAAAGAVVGGLG); its structural character is markedly influenced by several physiological factors such as ionic-strength, pH, and solvent composition [35,37]. This peptide readily forms amyloid fibrils that are resistant to proteinase K and pronase digestion [41], are neurotoxic, and induce the activation of astrocytes and microglial cells *in vitro* [42]. Conformational changes of PrP(106-126) as a function of pH are specifically confined to the alanine-rich peptide sequence, AGAAAAGA [43], and the valine-rich tail region, VVGGLGG. Gasset et al. [44] suggested that the valine-rich portion of the tail may have no role in determining the secondary structure of PrP(113-127). Various groups have established that prion amyloid propagation can have several different effectors – such as metallic ion imbalance and skewed metabolic products in the cells. These factors may work cooperatively to cause disease in the majority of cases. Accumulation of PrP^Sc^ appears to be spatially and temporarily linked with neurodegeneration and astrogliosis. Thus, neuronal death in prion-related encephalopathy might be due to the cell-aggregate interactions of PrP^Sc^ and/or its degradation products.

Here we conducted conformational studies on PrP(111-126) (HMAGAAAAGAVVGGLG), an amyloid-forming aggregation prone fragment. By doing so, we asked how different physicochemical conditions such as pH, membrane-like environments, and concentrations influence the conformation and self-assembly of PrP(111-126). Further, the binding of Cu^2+^ ions with PrP(111-126) and the probable effect of its concentration were analyzed. In order to examine the dynamics of structural transition, we employed spectroscopic techniques that are able to probe the solution-state structure of prion peptide fragments. We also studied the effects of PrP(111-126) on the viability of cultured rat astrocytes. It is possible that some segment of the prion assembles to initiate aggregation and stabilize the toxic conformations, thereby making the metal binding sites inaccessible for binding. Therefore, a detailed structural understanding of prion assemblies in various microenvironments and in the vicinity of metal ions is necessary to gather substantial information from amyloid toxicity.

## Results and Discussion

### Conformational preference of PrP(111-126) in aqueous solution

#### Time-dependent conformational changes

**Figure 1:**
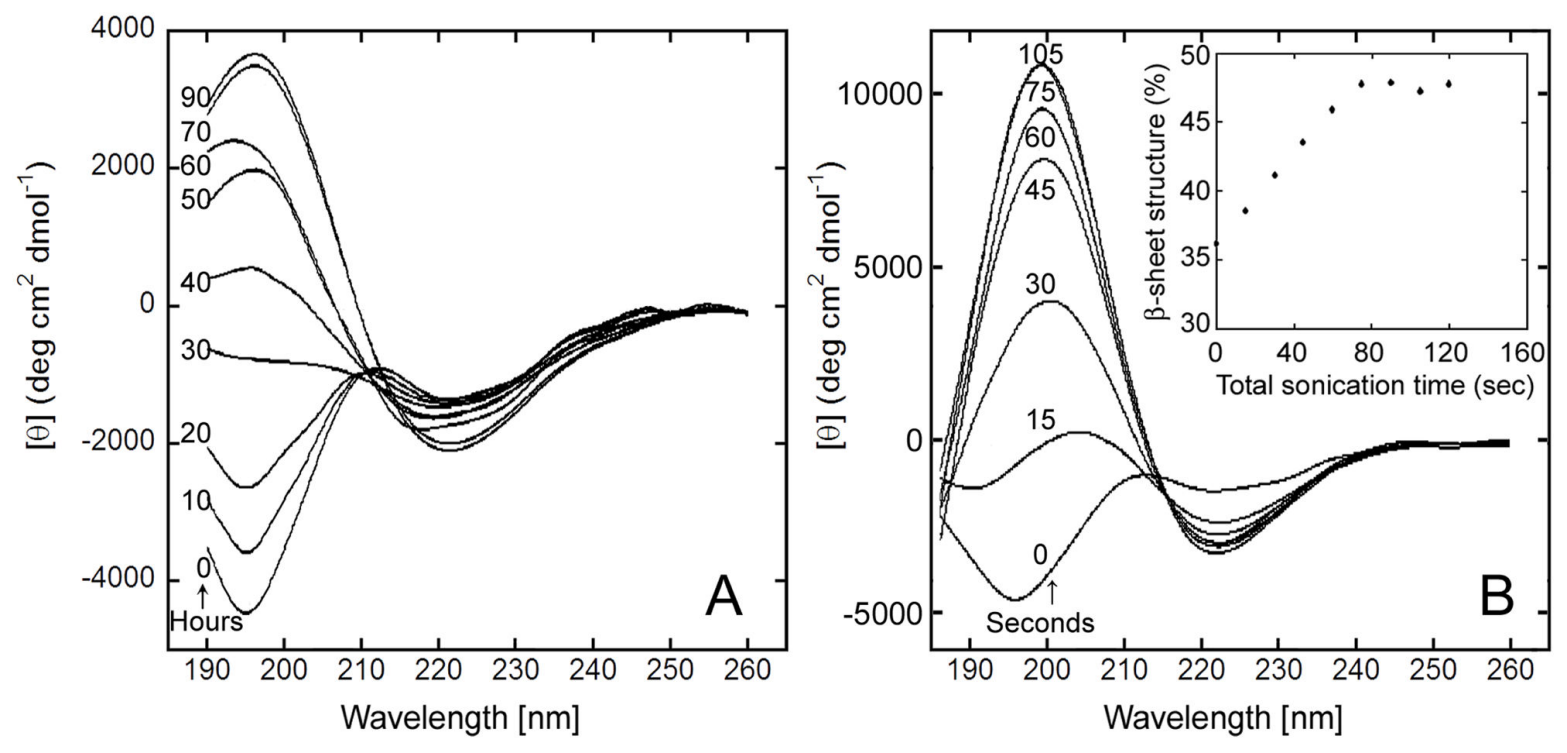
Time-dependent and sonication induced β-sheet formation of 20µM PrP (111-126). (A) CD spectra of PrP(111-126) over time in PBS at 37 °C. Spectra recorded at 0, 10, 20, 30, 40, 50, 60, 70 and 90 hours, (B) Sonication induced sheet formation in PrP(111-126). CD spectra of PrP(111-126) in PBS sonicated between measurements. Total sonication times are (in seconds) 0, 15, 30, 45, 60, 75 and 105. (inset) plot of % β- sheet structure versus total sonication time (sec).

As soluble, low molecular weight oligomers, full-length prion protein (PrP) reveals neurotoxicity both *in vitro* and *in vivo* (Simoneau et al., 2007). However, under *in vitro* conditions, fibrillar forms of PrP lack consistent toxicity. To investigate the self-assembly and aggregation of PrP(111-126), circular dichroism (CD) spectral experiments were performed. The CD spectrum of PrP(111-126) in phosphate buffered saline, pH 7.4 (PBS), (Figure 1A; curve a), indicate that PrP(111-126) adopted predominantly random coil conformation. Deconvolution of the spectrum using CONTIN program [45] showed the percentage of other components such as helix, β-sheet, and turn conformations (Table 1), with β-sheet content of ~36 percent. When incubated at 37°C, the PrP(111-126) solution showed an increase in the β-sheet structure over time, characterized by a more intense negative band at 222 nm and positive band at 198 nm, with a cross over near 212 nm (Figure 1A). A gradual loss of random coil structure over time, commensurate with the gain in β-sheet structure, was observed during the process of structural transition (Table 1). In the past, we have observed similar structural transitions for other amyloid forming peptides [35,46,47,48,49,50]. Such transitions are well-known in other amyloid aggregates that are associated with protein conformational diseases [51,52,53,54,55]. To understand the role of hydrophobicity, similar time-dependent control-experiments were performed on PrP(106-126) (KTNMKHMAGAAAAGAVVGGLG) and PrP(113-127) (AGAAAAGAVVGGLGG) (Figure S1). The results indicate that the time dependent conformational changes of both the peptides are similar and that the hydrophilic residues KTNMKHM of PrP(106-126) or HM of PrP(111-126) play negligible role in the aggregation process and the aggregation and β-sheet formation are mainly driven by the hydrophobic region of the peptide [55].

**Table 1:** Secondary structure changes in PrP(111-126) on aging.

Aging time (hours)	% secondary structure			
	α-Helix	β-Sheet	β-Turn	Random coil
0	4.6	36.2	20.8	38.4
18	4.2	37.1	21.2	37.5
33	4.7	38.2	20.5	36.6
40	4.5	38.9	20.8	35.8
44	4.3	39.8	21.1	34.8
49	4.1	40.7	21.3	33.9
60	4.2	41.6	21.2	33
70	3.9	42.9	20.9	32.3
90	4.1	42.8	21.3	31.8
110	3.9	43.1	21.4	31.6

#### Sonication-induced β-sheet Formation in PrP(111-126)

To investigate the stability of the initial random coil structure, the freshly dissolved (monomeric) peptide was subjected to sonication. CD spectrum of the initial PrP(111-126) solution was recorded and then sonicated for 15 seconds. The measurement was repeated with 15 seconds of sonication between each measurement, and the CD spectrum was recorded at 15-second intervals (Figure 1B; curves a - g). β-sheet content of PrP(111-126) was observed to increase over time as a result of sonication (Figure 1B; inset & Table 2). This indicates that sonication accelerates the coil to sheet structural transition. In the past, we have demonstrated similar behavior for PrP(113-127) peptide [35].

**Table 2:** Sonication induced changes in secondary structure of PrP (111-126).

Total sonication time (seconds)	% of secondary structure			
	α-helix	β-sheet	β-turn	Random coil
0	4.4	36.2	21.1	38.3
15	4.4	39.1	21.0	35.5
30	4.7	41.3	21.2	32.8
45	4.8	43.8	21.0	30.4
60	5.1	46.2	20.9	27.8
75	5.2	47.9	21.1	25.8
90	5.3	48.3	20.7	25.7
105	5.6	47.8	21.3	25.3
120	5.3	48.2	21.1	25.4

### Effect of microenvironment on the secondary structure of PrP(111-126)

Proteins experience various intra and extra cellular environmental conditions that can influence the structural changes that lead to toxic forms based on their conformational stability. To test the conformational stability and polymorphic behavior, PrP(111-126) was subjected to various microenvironments.

#### Influence of 1,1,1-trifluoroethanol (TFE) on PrP(111-126) Conformation

**Figure 2:**
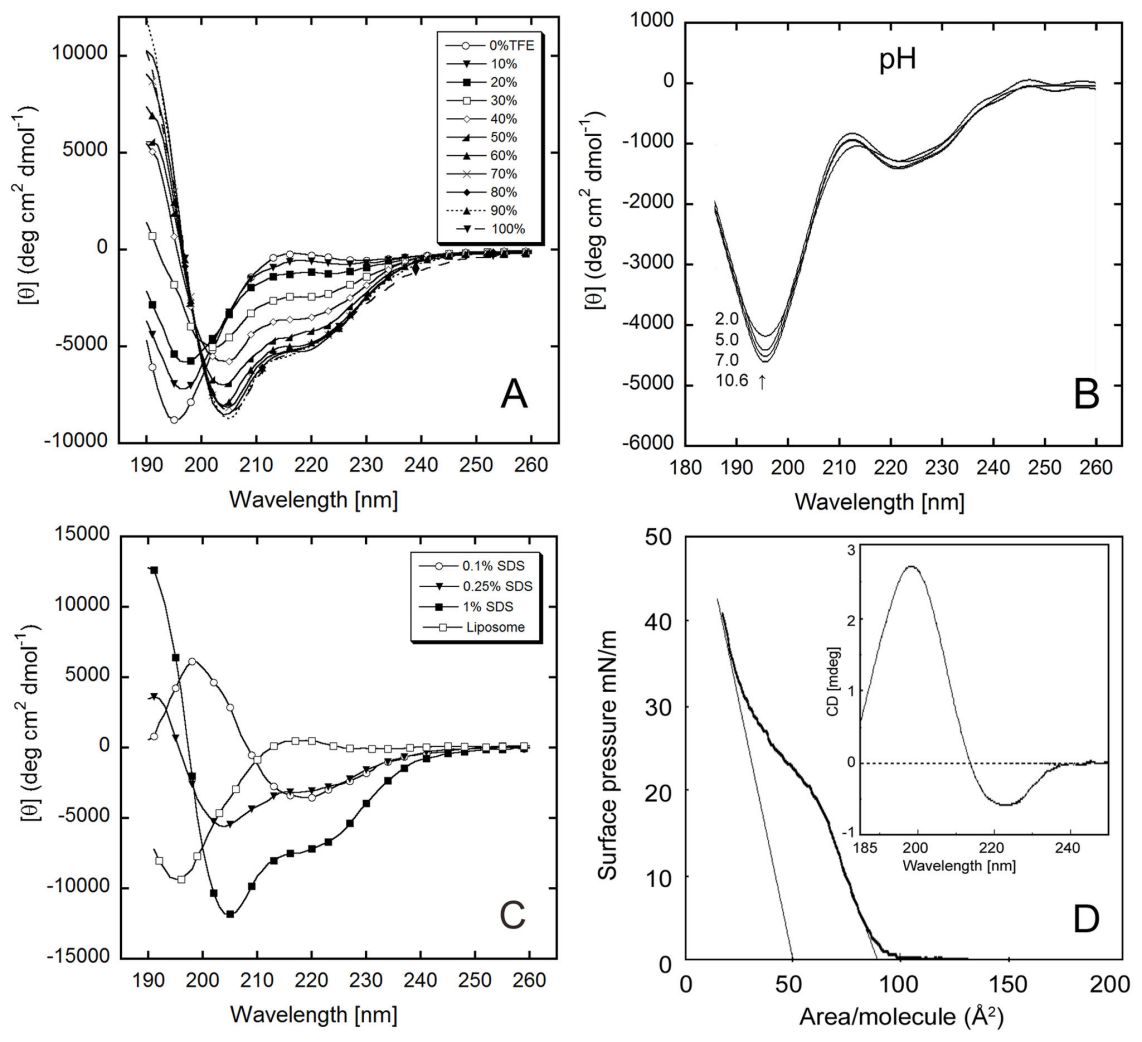
Effect of microenvironment on the secondary structure of PrP (111-126). (A) CD spectra of 20µM PrP(111-126) in different TFE concentrations, 0% to 100% TFE, in PBS at 4 °C. (B) CD spectra of PrP(111-126) at pH 2, pH 5, pH 7, and pH 10.6 at 20°C. (C) CD spectrum of PrP(111-126) in 0.1%(<CMC), 0.25%(CMC), 1.0%(>CMC) of SDS, and in liposomes in deionized water at 20 °C. (D) surface area-pressure isotherm of PrP(111-126) at air-water interface, and (inset) CD spectrum of peptide film (20 layers) on quartz showing β-sheet formation.

TFE facilitates α-helical formation in peptides, depending on the primary structure of the peptide [56,57,58]. Proteins containing non-helical segments have lower propensities for α-helical formation. According to the Chou-Fasman method for secondary structure prediction [59], the region homologous to the residues 113-126 has two major domains, an α-helical region favoring residues 113-118, and a β-sheet domain consisting of residues 119-126 [35]. This prediction indicates that PrP(111-126) has a tendency to exist with both α-helical and β-sheet formations. The concentration dependence of TFE for α-helix formation determines the stability of the conformation. To study the conformational stability, CD was performed. The CD spectra showed that the PrP(111-126) primarily had a random coil structure in aqueous solution, and an α-helical structure in 100% TFE (Figure 2A). The increase in α-helical structure was accompanied with a decrease in random coil structure. These results suggest that TFE stabilizes the helical structure of PrP(111-126). The results of titration using TFE as a co-solvent, which is consistent with the data reported from other groups, emphasize the crucial role of hydrophobic interactions in the structural transition of the peptide [60,61,62]. In summary, these results suggest that the solvent environment around the peptide backbone is an important determinant of peptide conformation.

#### Influence of pH on PrP(111-126) conformation

The conformations of prions are largely influenced by pH, as low pH triggers conversion of PrP^C^ to PrP^Sc^ [63]. To study the effects of pH on PrP(111-126), CD spectra were measured in aqueous buffers at pH 2.0, 5.0, 7.0 and 10.6 (Figure 2B). The results indicate a predominant random coil conformation at all pH conditions, suggesting a negligible role for electrostatic interactions in the self-assembly of this peptide, even though it contains a histidine residue and a methionine residue. This is consistent with the longer PrP(106-126) fragment, which bears ionic charges in the N-terminal region, and has been reported to show sheet conformation at pH 5, and less ordered conformation at pH 7 and further the hydrophilic fragment PrP(106-114) had no effect of pH [37,55]. Comparing these results signifies the importance of the hydrophobic segment toward β-sheet formation in the PrP(106-126) at pH 5. Given these results, combined with the fact that the secondary structure of PrP(111-126) is essentially independent of ionic strength, it seems that electrostatic interactions play a negligible role in PrP aggregation. 

#### Surfactant Induced Conformational Changes in PrP(111-126)

Sodium dodecyl sulfate (SDS) is an anionic surfactant used to denature proteins by disrupting electrostatic interactions and incorporating hydrophobic portions of peptides into the hydrophobic regions of SDS micelles. The hydrophobic environment provided by SDS is used widely as a simple model for biomembranes, which can induce various conformations for amyloid peptides [64]. To study the effects of this microenvironment, PrP(111-126) was introduced into aqueous SDS solution at different concentrations. The CD spectra (Figure 2C) show that PrP(111-126) adopts a β-sheet conformation in 0.1% (w/v) solution (below the critical micellar concentration(CMC) of SDS), and a α-helical conformation at (0.25%) and above (1% w/v) CMC. This observation is similar to the helical conformation found in PrP(106-126), which indicates that the KTNMK sequence at the N-terminal region has a very small effects on the interactions with SDS [55]. When introduced to liposomal solution, PrP(111-126) showed a random coil conformation. The hydrophobic sequence of AGAAAAGAVVGGLG, PrP(113-126), offers a suitable condition for interacting with membranes, and has frequently been found in transmembrane proteins that are active in translocation [65,66], and a transition toward β-sheet conformation has been observed in signal peptides in a highly hydrophobic environment [67]. The environment-dependent structural transition leads us to surmise that the PrP^C^ PrP^Sc^ transition, which occurs in a post-translational process, may be driven by the environment around the PrP^C^ peptide. 

#### Langmuir isotherm and conformation of PrP(111-126) LB film

Observations of PrP(111-126) at the air-water interface indicated that stable films were formed. Figure 2D shows a pressure-area isotherm for an air-water interfacial PrP(111-126) film with two phases. This isotherm shows that the film of PrP(111-126) was a fairly rigid liquid-expanded type (85 Å^2^) film with its natively folded conformation. On further compression the molecules make a structural transition into a more packed structure. The value for the molecular area of the monolayer at the onset of the condensed phase was found to be 50 Å^2^ per molecule, and the collapse pressure was approximately 42 mN/m^2^. The CD spectrum of the transferred film (20 layers) is shown in the inset of Figure 2D. From the spectrum, it was determined that a PrP(111-126) film at the air-water interface predominantly adopts a β-sheet conformation. This indicates that there was a profound effect on β-sheet assembly that arose from oriented molecular crowding, and infers that, in contrast to the initial bulk solution containing high random coil and low β-sheet structures, a stable β-sheet enriched state of the PrP amyloid was produced by the effects of hydrophobic interaction at the air-water interface.

### Effect of Copper (II) on PrP(111-126)

**Figure 3:**
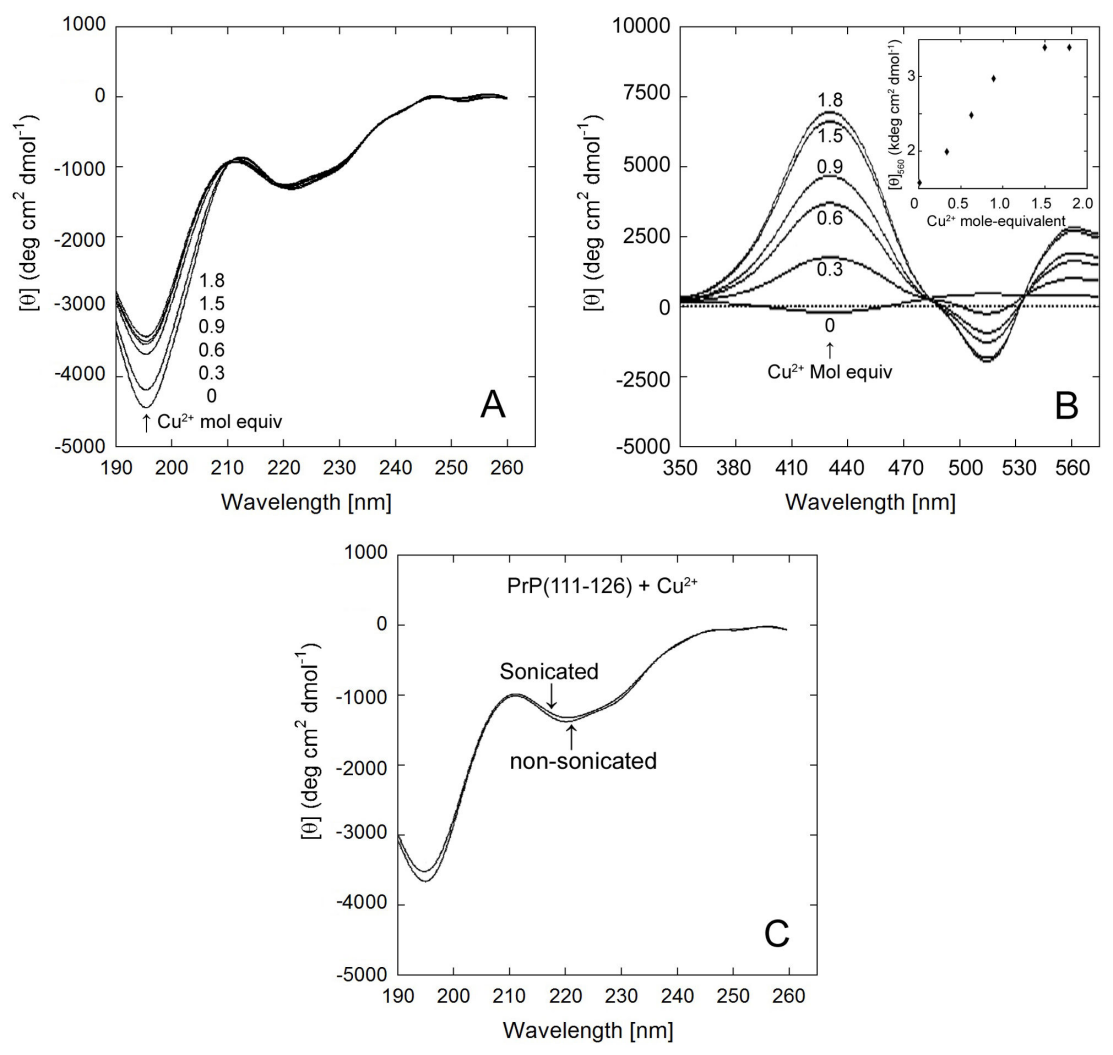
CD spectra of 20 µM PrP(111-126) with varying Cu^2+^ concentrations (0, 0.3, 0.6, 0.9, 1.5, 1.8 mol. equiv) in PBS at 20 °C. (A) UV-CD and (B) visible-CD spectra of PrP(111-126) with 0 to 1.8 mole equivalent of Cu^2+^. (B, inset) intensity of 560 nm CD band vs. Cu^2+^ concentration. (C) CD spectra of PrP(111-126) with or without sonication for 120 seconds in the presence of 1 mole equivalent Cu^2+^.

The binding of copper and other metal ions to PrP^C^ have been suggested to play a vital role in the misfolding process of PrP^C^. An earlier work denotes suppression of PrP(106-126) fibrils in the presence of Cu^2+^ ions [68]. In particular, histidine residues are known to promote ion mediated structural transition. Hence, this study explores the conformational preferences of the PrP(111-126) fragment with respect to copper ion concentration and related microenvironments. The CD spectrum of PrP(111-126) in the absence of Cu^2+^ contained negative bands at 196 nm and at 222 nm (Figure 3A). As CuSO_4_ was titrated into the peptide solution, the band intensity at 196 nm decreased slightly (Δθ = 680 deg.cm^2^.dmol^-1^), indicating some effect on random coil structure. The variation in the random coil structure on the addition of Cu^2+^ suggests that the histidine residue may be present at the random coil region of PrP(111-126). CD spectra of metal-bound proteins were observed only in the visible wavelength region. CD bands were observed at 560 nm and 430 nm on addition of Cu^2+^ to PrP(111-126) (Figure 3B). These CD bands increased with an increasing concentration of Cu^2+^ up to 1 mole-equivalent. Further addition of Cu^2+^ showed no effect on the CD spectra, suggesting that the stoichiometric ratio of Cu^2+^ binding to PrP(111-126) is 1:1. The inset of Figure 3B shows a plot of the CD band intensity at 560 nm versus Cu^2+^ mole-equivalent of PrP(111-126). The K_d_ of binding for Cu^2+^ to PrP(111-126) was computed to be ~ 4.5 μM (at 0.021 mM peptide). 

Previous studies provide evidence that the binding sites for Cu^2+^ to the peptide, and to the full length protein, are the same [69,70,71]. In mammalian cells, PrP^C^ is located at the outer cell surface where it is fixed to the plasma membrane by a glycosylphosphatidylinositol anchor at its C-terminus, and thus is free to bind extracellular Cu^2+^. A physiologic role of PrP/Cu^2+^ binding [72] has been supported by studies showing lowered levels of Cu^2+^ in sub cellular fractions taken from the brains of PrP deficient mice [73]. Proteins that bind to Cu^2+^ are candidates for the sensing and/or transport of copper. Sonication-induced sheet formation, demonstrated for a pristine prion solution, was not observed for PrP(111-126) in the presence of 1 mole-equivalent of Cu^2+^ (Figure 3C). These results suggest that Cu^2+^ not only binds to PrP(111-126), but that it also inhibits the aggregation of PrP(111-126). Copper bound PrP(111-126) is more stable and prevents sheet formation and misfolding.

### Morphology of PrP(111-126) assemblies

**Figure 4:**
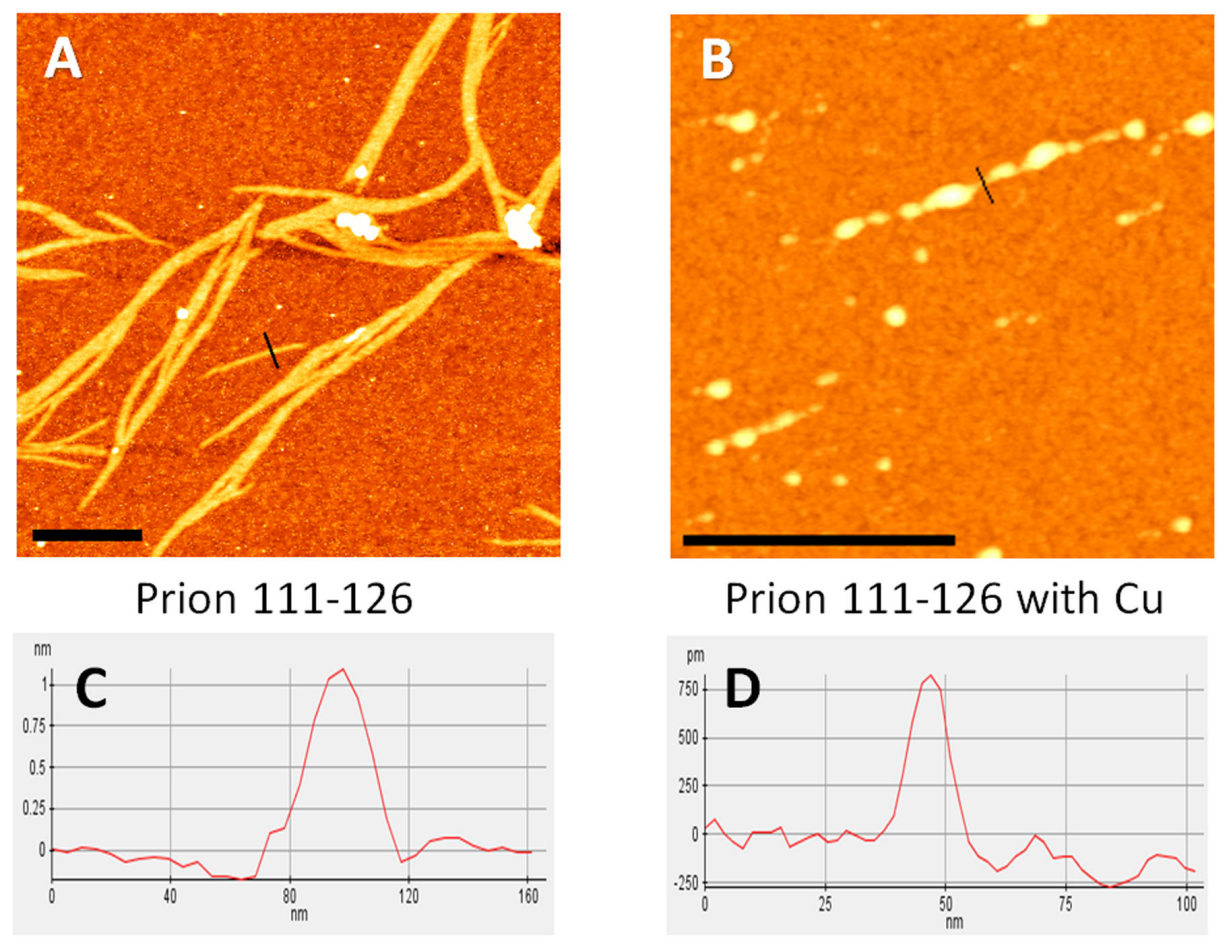
Morphology of fibrillar assemblies of the PrP (111-126). AFM height images of (A) PrP(111-126) and (B) PrP(111-126) in the presence of Cu^2+^ ions. Height profiles of the fibrils for lines marked on the images: (C) PrP (111-126) and (D) PrP (111-126) with Cu^2+^. Scale bar represents 500nm.

Morphology of self-assembled PrP(111-126) peptide with and without copper ions was studied by atomic force microscopy (AFM) (Figure 4). To the best of our knowledge, this is the first AFM study uncovering Cu^2+^ ion’s effects towards PrP(111-126) aggregation. The image (Figure 4A) shows the fibrillar assemblies of the peptide that are often grouped together in bundles. The length of the fibrils ranged from 1micron to 7 microns. Individual fibers are narrow stripes of approximately 5 nm in width. The length of single fibers is about 500 nm and the height of individual fibers and aggregates in the form of stripes is 1 nm. The maximum roughness of the top surface of the stripes is less than 0.1 nm across the stripe (Figure 4C). The width of combined prion fragment sheets reaches 60 nm or more. In the presence of copper ions, the peptides formed globular structures that were often connected together like a beaded chain (Figure 4B). The size of each globular assembly ranged from 0.1 to 1.5 microns or greater and the minimum height of the linkage between each aggregates was ~ 0.75 nm (Figure 4D). This indicates that copper ions prevent fibril formation and promote globular aggregates by binding to the most probable histidine site that is present at the N-terminal region of the peptide. PrP(113-127) formed fibrils in the presence and in the absence of the copper ions (Figure S2). Prion proteins lacking a histidine residue have been shown to be neuroprotected from copper toxicity [74]. Further mutagenesis of His-111 seems to break the neurotoxicity of PrP(106-126) [68]. Copper ion binding to the imidazole side chain of histidine is well-known, and our results for PrP(111-126) are consistent with other prion peptide sequences containing histidine [68,75,76].

### Toxicity Studies

**Figure 5:**
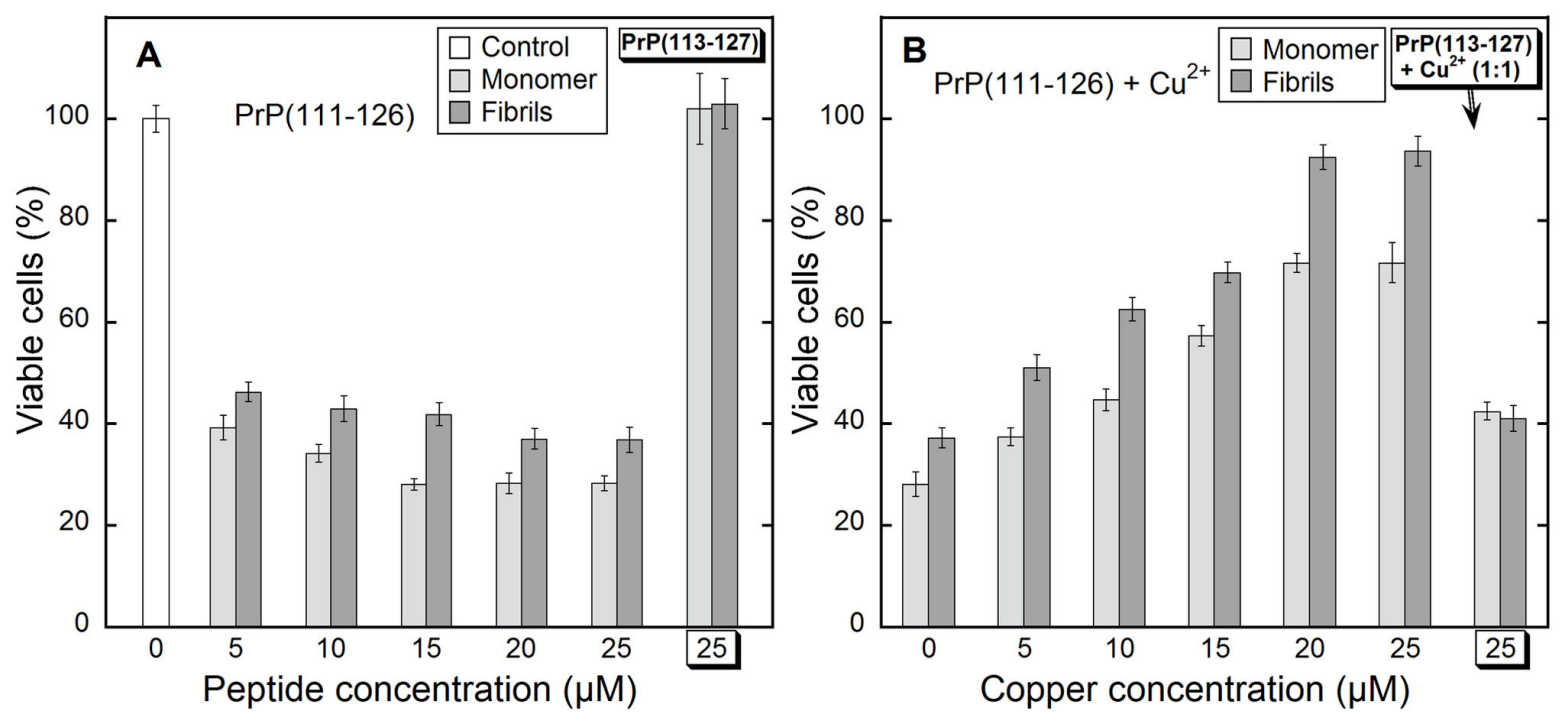
Cell viability (MTT) assay. Monomeric and fibrillar form of PrP(111-126) and PrP(113-127) were treated to astrocyte cultures. (A) Cytotoxicity of monomers and fibrils at various concentrations of PrP(111-126) and at 25µM of PrP(113-127). (B) PrP(111-126) (20µM) in the presence of different concentrations of Cu^2+^and 25µM of PrP(113-127) with 25µM Cu^2+^.

Cytotoxicity of PrP(111-126) in astrocyte cultures was examined by MTT assay. Cells were incubated with monomeric and fibrillar forms of PrP(111-126) at various concentrations of the peptide. The results indicated that both forms of PrP(111-126) were toxic, however monomers were relatively more cytotoxic than fibrils (Figure 5A). The toxicity of the monomers may be due to the co-existence of low-order oligomeric aggregates, which are known to be more toxic than fibrillar aggregates[77,78]. Toxicity was dose-dependent, increasing up to 15 µM, and thereafter remaining constant at higher concentrations. Toxicity of monomers (20µM) or fibrils (20µM) was evaluated with different Cu^2+^ concentrations. A significant and dose-dependent reduction in PrP mediated toxicity was observed when treated with Cu^2+^ bound peptide (Figure 5B). These results are consistent with the observed non-fibrillar morphology of the peptide aggregates in the presence of copper ions (Figure 4B). To study the role of histidine, the toxicity experiments were carried out using monomeric and fibrillar PrP(113-127), which contain no histidine. Both (monomeric and fibrillar) forms of PrP(113-127) were found to be non-toxic (Figure 5A). Mutating histidine residue to alanine or serine residues, in neurotoxic prion peptide sequences, has also been shown to be non-toxic [68,74]. Further, the addition of Cu^2+^ to PrP(113-127) showed toxicity that may be due to the presence of unbound copper ions (Figure 5B). Therefore, the toxicity of PrP(111-126) may be due to the chelation of the cellular metal ions by the histidine residue, as the copper-bound peptide was nontoxic [68,75,76]. The prion peptides can also act as an antioxidant, enhancing the survival of the astrocytes by binding potentially harmful Cu^2+^ ions and by quenching the free radicals generated as a result of copper redox cycling [79]. It appears that copper mediated NMDAR activation by prions could ameliorate amyloid toxicity by reducing glycine receptor sensitization. Therefore, it is probable that agents that would chelate copper ions can adversely increase the neurotoxicity of prions [80].

## Summary and Conclusions

The prion protein fragment PrP(111-126), a hydrophobic hexadecapeptide, adopts predominantly random coil conformation under aqueous conditions, regardless of pH levels. TFE as a cosolvent induced α-helical conformation. An external stress from either sonication or denaturing SDS resulted in β-sheet conformation. At the air-water interface, it formed a stable monolayer and acquired β-sheet conformation in a packed state. These conformational preferences and structural transitions may occur due to the intrinsic hydrophobic-hydrophilic nature of the system that generates various microenvironments for the peptide. Electrostatic interactions appeared to play a negligible role during aggregation of PrP(111-126). By comparing all of the above variables in PrP microenvironment, the major driving forces for PrP(111-126) to self-assemble appear to be greater hydrophobicity and intermolecular hydrogen bonding that results in β-sheet formation. 

PrP(111-126) bound to Cu^2+^ at a stoichiometric ratio of 1:1 via histidine preserved its secondary structure. Most studies agree that at least two free copper binding sites (96 and 111) are present in PrP. Cytotoxicity assays of PrP with astrocyte cells showed that both monomeric and fibrillar forms of PrP were toxic to astrocytes, and the presence of copper decreased the toxicity. In cells, PrP^C^ is located at the outer surface, fixed to the plasma membrane where it is free to bind extracellular Cu^2+^. Thus, PrP may play an important role in the metabolization of copper. Our studies indicated a crucial role of histidine in self-assembly, aggregation, and toxicity.

## Experimental Procedures

### Materials

All reagents used in peptide synthesis and other experiments were of the purest analytical grade; t-Butyl carbazate, 1-Hydroxy benzotriazole (HOBt), N,N’-diisopropylcarbodiimide (DIC), trifluoromethane sulphonic acid (TFMSA), Phenyl isothiocyanate (PITC), ethanedithiol, thioanisole, dichloromethane (DCM), 1,1,1-trifluoroethanol (TFE) and trifluoroacetic acid (TFA) were purchased from Aldrich, Germany. SDS was obtained from SRL, India.

### Peptide Synthesis and Purification

The peptide PrP(111-126) was synthesized by solid phase peptide synthesis (SPPS), using Wang resin beads and Fmoc as the protective group for N-terminal ends. Fmoc-amino acids were prepared using standard procedures [81] and were characterized by thin layer chromatography and FTIR spectroscopic studies. Deprotection was accomplished using piperidine, and activation of the carboxylic ends of the amino acids for coupling was done using 1-Hydroxy benzotriazole (HOBt), and N,N’-diisopropylcarbodiimide (DIC). Initiation and growth of the peptide formation was performed on Wang resin with an alkoxybenzyl alcohol functionalized surface, with the initial amino acid anchored to the resin via the formation of a benzyl ester. All steps for initiation, deprotection, activation, and coupling were performed in DMF. Following construction of the peptides, they were cleaved from the resin with a [1:1:1:7] mixture of TFMSA / thioanisole / 1,2-ethanedithiol / TFA [82], then precipitated with cold ether. The composition of peptides was determined by amino acid analysis using PITC method. 

### Sample preparation

The PrP peptide was disaggregated via the TFA/TFE method [83]. Briefly, PrP(111-126) was disaggregated by pretreatment with TFA followed by treatment with TFE three times to remove traces of TFA. After each step, solvents were evaporated to form a thin film in the tube. The film was dissolved in phosphate buffered saline, (PBS), pH 7.4, and used immediately for the experiments.

### Circular Dichroism (CD) Spectroscopy

CD spectra were measured in a quartz cell with an optical path of 0.1 cm, using a Jasco J-715 (Tokyo-Japan) spectropolarimeter at a scan speed of 50 nm/min. The percentages of the secondary structures of PrP(111-126) were calculated using the SELCON3 software program [45]. For the TFE titration experiments, stock solutions of the PrP peptides were prepared in a phosphate buffer (50 mM, pH 7) and in neat TFE. The two stock solutions were mixed to provide peptides in concentrations of 0, 5, 10, 15, 20, 25, 30, 40, 50, 60, 70, 80, 90, 95, and 100% TFE. CD spectra were recorded at a temperature of 4°C. To analyze the effects of SDS micelles on the PrP(111-126) conformation, CD spectra were collected from solutions of PrP in 0.1%, 0.25% and 1% SDS in deionized water. Liposomes were prepared using DSPC/ Cholesterol/PEG(3400)-DSPE (59:29:12, w/w) and the ratio of liposomes to peptide was 30:1(w/w). 

### Langmuir-Blodgett film Formation

Langmuir-Blodgett (LB) films of 2 mmol solutions of PrP(111-126) in 10% methanol in chloroform were prepared using a Nima trough 611 with a Wilhelmy balance. A micropipette was used to spread each PrP solution across the maximum available area of an aqueous sub-phase (filtered water from MQ Millipore with resistivity of 18 MΩ). Hydrophilic quartz slides and surface modified hydrophobic quartz slides [84] were coated with LB films of PrP, and used for spectroscopic measurements. The organic solvent was allowed to evaporate and the monolayer was compressed until a desired surface pressure was reached. These monolayers were then transferred to quartz slides by y-type deposition (20 layers). 

### AFM measurements

AFM measurements were made using an NX10 instrument from Park Scientific, South Korea, with moderately stiff cantilevers (k ~5 N/m at low levels of cantilever oscillation, damping less than 30%). Imaging was performed on PrP(111-126) and PrP(113-127) in the absence and presence of Cu^2+^ ions. A solution of 0.3mM of PrP(111-126)/(113-127) in PBS (with and without Cu^2+^) was incubated at 37°C for 5 days. Samples were diluted 1:20 with deionized water and 10 µl of the solution was placed on a silicon wafer and evaporated under vacuum. 

### Cell Culture

Astrocyte cultures were prepared from the cerebral cortices of 2-day-old neonate Wistar rats according to published protocols [85,86]. Briefly, dissociated cells were suspended in modified Eagle's medium containing 30 mM glucose, 2 mM glutamine, 1 mM pyruvate and 10% fetal bovine serum, and were plated on an uncoated 25 cm^2^ flask at a density of 6,000,000 cells/cm^2^. A monolayer of type I astrocytes was obtained 12-14 days after the plating. Non-astrocytes such as microglia were detached from the flasks by shaking, and then removed by changing the medium. Astrocytes were dissociated by trypsinization and reseeded on uncoated 96-well plates at a density of 1,000,000 cells/cm^2^. Cells were used for experiments after they became confluent (approximately 7-8 days later). The surgical procedure was performed in a laminar flow hood under aseptic conditions following NIH animal ethical guidelines and following approval by the animal care committee of Central Leather Research Institute, Chennai, India.

### MTT Assay

The MTT assay is based on the ability of a mitochondrial dehydrogenase enzyme from viable cells to reduce 3-(4,5-dimethylthiazol-2-yl)-2,5-diphenyltetrazolium bromide (MTT), and form dark blue formazan crystals which are largely impermeable to cell membranes, resulting in formazan accumulation in healthy cells. Solubilization of the cells by the addition of dimethylsulfoxide (DMSO) results in the liberation of the crystals, which are also solubilized. The number of surviving cells is directly proportional to the level of the formazan product created. For cytotoxic experiments, astrocytes were plated in 96-well trays. After PrP peptide treatment, cells were removed and a MTT assay was carried out as described by Fezoui et al. [87]. Two assays were performed using fibrils (pre-incubated) and freshly dissolved (not pre-incubated). For fibril preparation, prion peptides were pre-incubated at a concentration of 2 mM in 20 mM HEPES buffer, pH 7.4, at 37°C for seven days. They were then diluted in 100 μl of DMEM medium and placed in 96-well microtitre plates to yield final PrP(111-126) or PrP(113-127) peptide concentrations of 5, 10, 15, 20, and 25 μM, to which astrocytes were added. To study the effect of copper ions on toxicity, copper solution was mixed with the monomeric and fibrillar peptides (1:1 mole ratio). This mixture was then added to the astrocytes without incubation. A freshly prepared solution of PrP(111-126) peptide was also added to culture DMEM medium without pre-incubation, and placed in 96-well microtitre plates, to yield final PrP(111-126) peptide concentrations of 5, 10, 15, 20, and 25 μM, to which astrocytes were added. A filtered sterile stock solution of 5 mg MTT per ml of PBS was added directly to each well to give a final concentration of 0.5 mg/ml. Cell survival was determined after 48 hours. The MTT formazan product was released from cells by addition of dimethyl sulfoxide (DMSO) and measured at 570 nm. The relative survival of cells was determined by comparing results with untreated control cell cultures. MTT (cell culture grade) and DMSO were obtained from Sigma, Germany.

## Supporting Information

Figure S1 **CD spectra showing time-dependent β-sheet formation of 20µM PrP(106-126) and PrP(113-127) in PBS at 37 °C.**
(TIF)

Figure S2 **AFM images of PrP(113-127) showing the morphology of fibrillar assemblies of the PrP(113-127) in the absence and presence of Cu^2+^ ions.**
(TIF)
