# Supplementary figures and images for: Solvent Microenvironments and Copper Binding Alters the Conformation and Toxicity of a Prion Fragment
Source: PLoS One. 2013 Dec 27;8(12):e85160. doi: 10.1371/journal.pone.0085160 (PMC3874036; doi:10.1371/journal.pone.0085160)

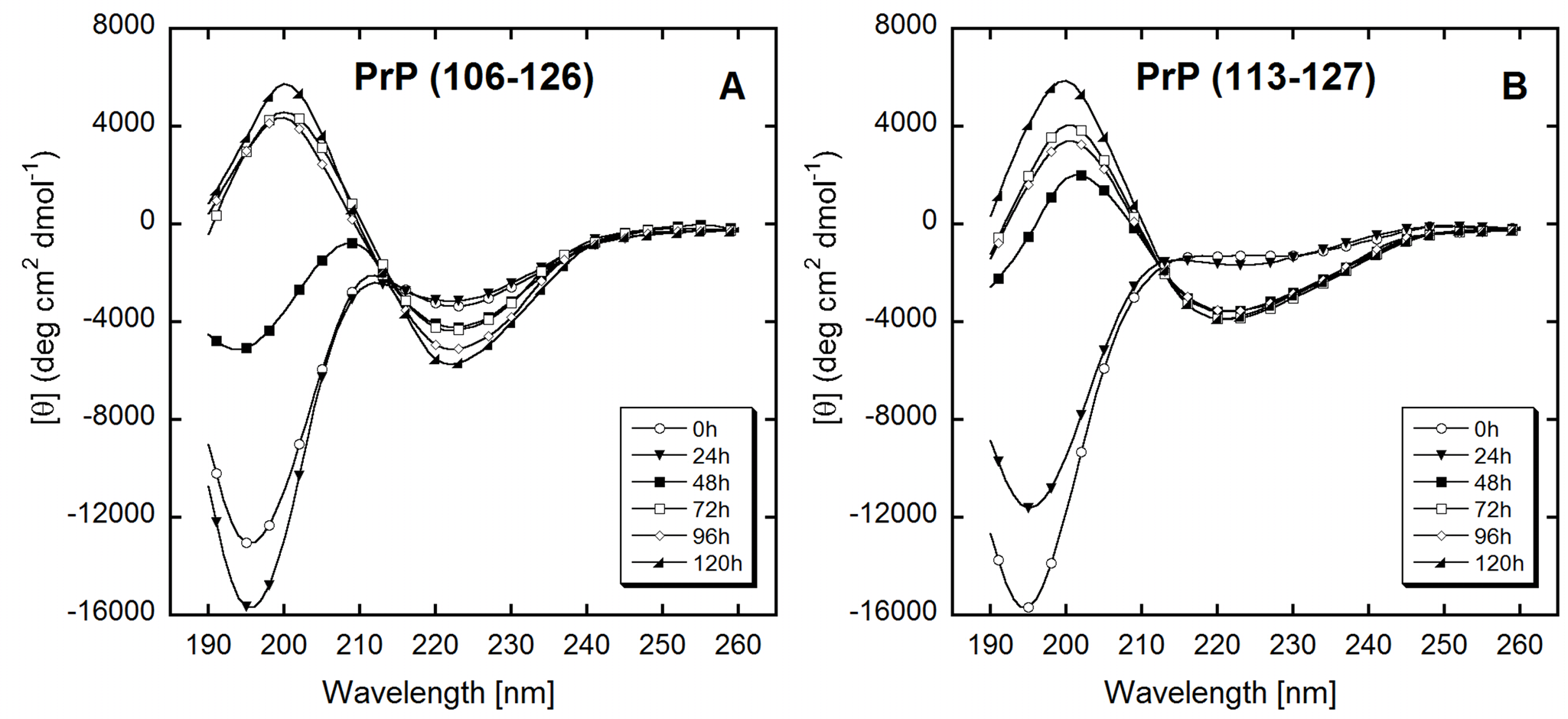

Supplement: Figure S1 — CD spectra showing time-dependent β-sheet formation of 20µM PrP(106-126) and PrP(113-127) in PBS at 37 °C. (TIF) [file pone.0085160.s001.tif]

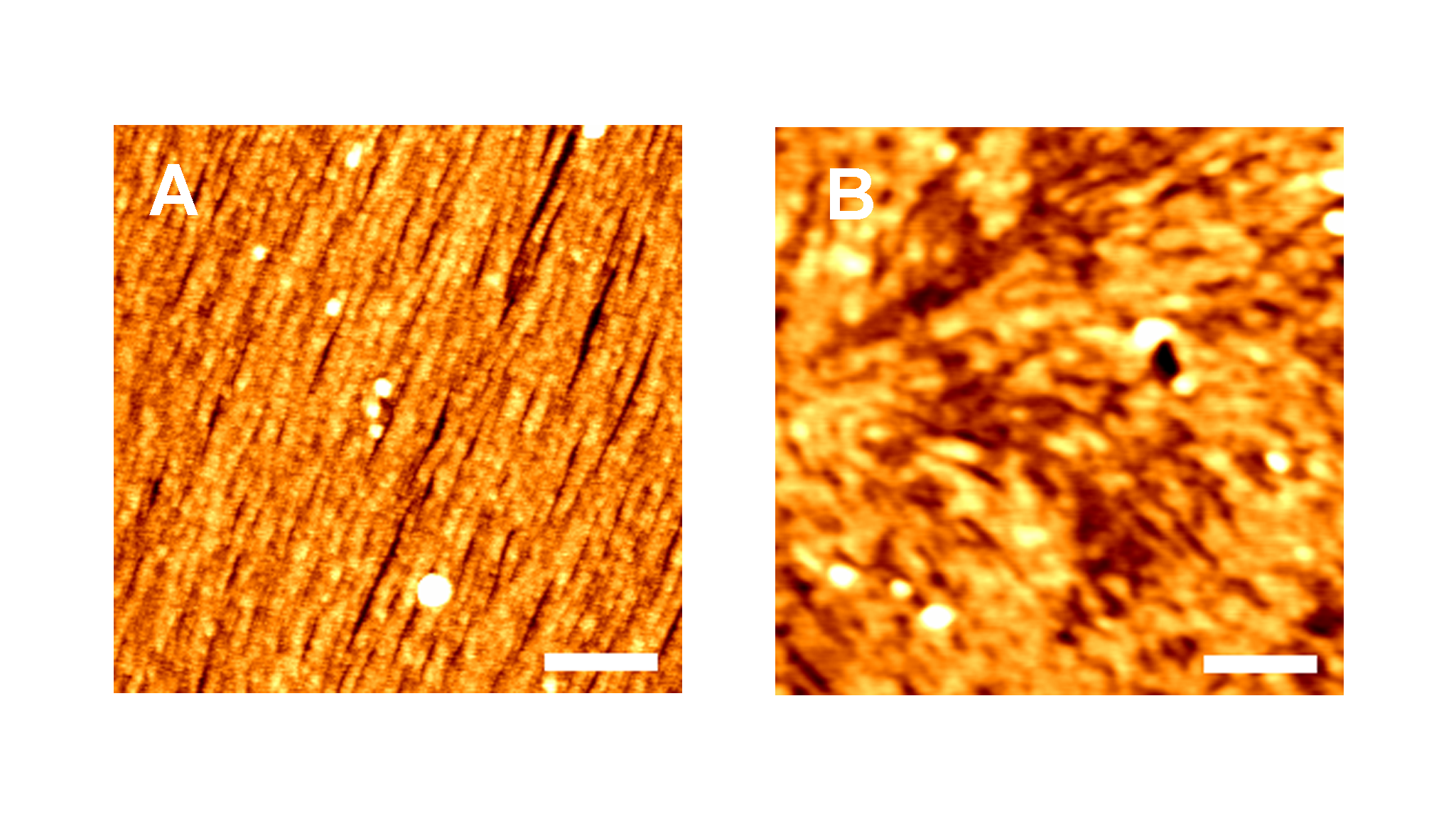

Supplement: Figure S2 — AFM images of PrP(113-127) showing the morphology of fibrillar assemblies of the PrP(113-127) in the absence and presence of Cu2+ ions. (TIF) [file pone.0085160.s002.tif]
